# Pre-existing subclinical hypothyroidism and low free thyroxine in patients undergoing immune checkpoint inhibitor therapies is a risk factor for overt hypothyroidism

**DOI:** 10.3389/fendo.2025.1690196

**Published:** 2025-11-10

**Authors:** Yu Bai, Yue Yin, Hong Liu

**Affiliations:** Key Laboratory of Carcinogenesis and Translational Research (Ministry of Education), Department of Pharmacy, Peking University Cancer Hospital and Institute, Beijing, China

**Keywords:** immune checkpoint inhibitors, thyroid dysfunction, abnormal baseline thyroidfunction, hypothyroidism, immune-related adverse events

## Abstract

**Background:**

Immune checkpoint inhibitors (ICIs) have transformed cancer therapy. However, these therapies commonly precipitate immune-related adverse events, with thyroid dysfunction being the most frequent manifestation of endocrine toxicity. Whether subclinical abnormal baseline thyroid function (sABTF) predisposes to overt thyroid dysfunction during ICI therapy—and if so, how it influences time-to-onset, phenotype, severity, and clinical decision have not been fully elucidated.

**Methods:**

We conducted a retrospective cohort study of 235 patients with cancer who underwent ICI therapy at Peking University Cancer Hospital between January 1, 2018, and September 30, 2024. Patients were stratified by baseline thyroid function status and monitored for ICI-induced overt thyroid dysfunction. Multivariate logistic regression analysis identified the risk factors for overt thyroid dysfunction. To examine baseline free thyroxine (FT4) cutoff, we used restricted cubic splines and receiver operating characteristic (ROC) analysis. Time-to-event was estimated using Kaplan-Meier curves and multivariate Cox models.

**Results:**

Among 99 patients with sABTF, 51.52% developed overt thyroid dysfunction during ICI therapy. Baseline FT4 was associated with overt hypothyroidism (adjusted odds ratio [OR], 0.88; 95% confidence interval [CI], 0.78–0.99; *p* = 0.037). A data-driven FT4 threshold of 13.91 pmol/L showed moderate discrimination (area under the ROC curve, 0.687). Using this threshold, higher FT4 (≥13.91 pmol/L) was associated with substantially lower odds of overt hypothyroidism (adjusted OR, 0.17; 95% CI, 0.06–0.47; *p* = 0.001), indicating that patients with FT4 <13.91 pmol/L were at markedly elevated risk. Severe subclinical hypothyroidism at baseline (TSH ≥10 mIU/L) was associated with a shorter time to overt hypothyroidism (median 7.78 weeks; adjusted hazard ratio [HR], 2.84; 95% CI, 1.42–5.67; *p* = 0.003), with the highest risk observed among patients who also had FT4 <13.91 pmol/L (median 6.57 weeks; adjusted HR, 10.05; 95% CI, 3.13–32.23; *p* < 0.001).

**Conclusions:**

Pre-existing thyroid abnormalities, particularly severe subclinical hypothyroidism with concurrent FT4 <13.91 pmol/L, identify a high-risk subgroup prone to an earlier onset of ICI-induced overt hypothyroidism. These data support comprehensive baseline assessments and intensified early monitoring to enable timely detection and management during ICI therapy.

## 1 Introduction

Immune checkpoint inhibitors (ICIs) have substantially improved outcomes across multiple advanced malignancies; however, these therapies increase the risk of immune-related adverse events (irAEs). Thyroid dysfunction is the most frequently reported manifestation of endocrine toxicity (1, 2). The estimated incidence of ICI-induced thyroid dysfunction ranges from 40% to 50%, with overt disorders, including hypothyroidism and thyrotoxicosis, occurring in up to 20% of patients during treatment (3, 4).

Most of the clinical studies and trials investigating ICI-related toxicity have primarily focused on patients with normal baseline thyroid function (NBTF). However, a significant subset of patients present with subclinical abnormal baseline thyroid function (sABTF) before treatment, including subclinical hypothyroidism, subclinical hyperthyroidism, or isolated abnormalities in free triiodothyronine (FT3) and free thyroxine (FT4). These individuals are frequently excluded from clinical trials and often overlooked in real-world studies. Previous studies have suggested that approximately 21% of patients exhibit abnormal baseline thyroid-stimulating hormone (TSH) levels (5, 6), and that among such patients, thyroid function frequently deteriorates following ICI initiation (median time to new or worsening dysfunction 6.6 and 4.7 weeks, respectively) (5). Therefore, more intensive monitoring is warranted during the first 8 weeks of therapy (5). Moreover, baseline TSH abnormalities have been associated with poor overall survival (6).

Clinical practice guidelines uniformly recommend screening for thyroid function before ICI initiation (7). Major medical societies advise periodic monitoring during treatment. The American Society of Clinical Oncology and Society for Immunotherapy of Cancer recommend monitoring function every 4–6 weeks (1, 2), and the European Society for Medical Oncology and European Society of Endocrinology recommend screening during each treatment cycle (7, 8). However, to our knowledge, specific management protocols for patients with sABTF have not yet been developed. Current guidelines typically recommend repeat TSH and FT4 testing at regular intervals or shared care with endocrinology (1, 2, 7, 8). Research in this area is limited, and the clinical characteristics of the patients with sABTF need to be systematically investigated to develop evidence-based management strategies.

To address this research gap, we conducted this retrospective cohort study to characterize the clinical course of ICI-induced overt thyroid dysfunction in patients with sABTF. Specifically, we compared the incidence and time to onset of overt dysfunction between those patients with and those without baseline abnormalities and explored risk stratification to identify clinically actionable high-risk subsets. We hypothesized that pre-existing thyroid dysfunction confers both a higher risk and an earlier onset of overt thyroid dysfunction after ICI initiation, particularly among high-risk subgroups.

## 2 Materials and methods

### 2.1 Study population

This retrospective study was conducted at Peking University Cancer Hospital between January 1, 2018, and September 30, 2024. Eligible patients were identified using the hospital’s electronic medical record system, based on both medication prescriptions and disease diagnosis. The administered ICIs included pembrolizumab, camrelizumab, tislelizumab, atezolizumab, nivolumab, sintilimab, toripalimab, durvalumab, serplulimab, ipilimumab, and cadonilimab. In addition, patients were required to have a diagnosis of thyroid dysfunction, manifested as hyperthyroidism, hypothyroidism, or thyrotoxicosis.

A total of 465 patients were initially identified. The inclusion criteria were as follows (1): age ≥18 years (2); thyroid function tests performed at baseline, defined as within 30 days before or up to 7 days after the initiation of ICI therapy; and (3) availability of thyroid function assessments during and after ICI treatment. The exclusion criteria were as follows (1): significant missing clinical data (2); presence of overt thyroid dysfunction at baseline in patients classified as having sABTF; and (3) absence of ICI-induced overt thyroid dysfunction in patients with normal baseline thyroid function.

**Figure 1:**
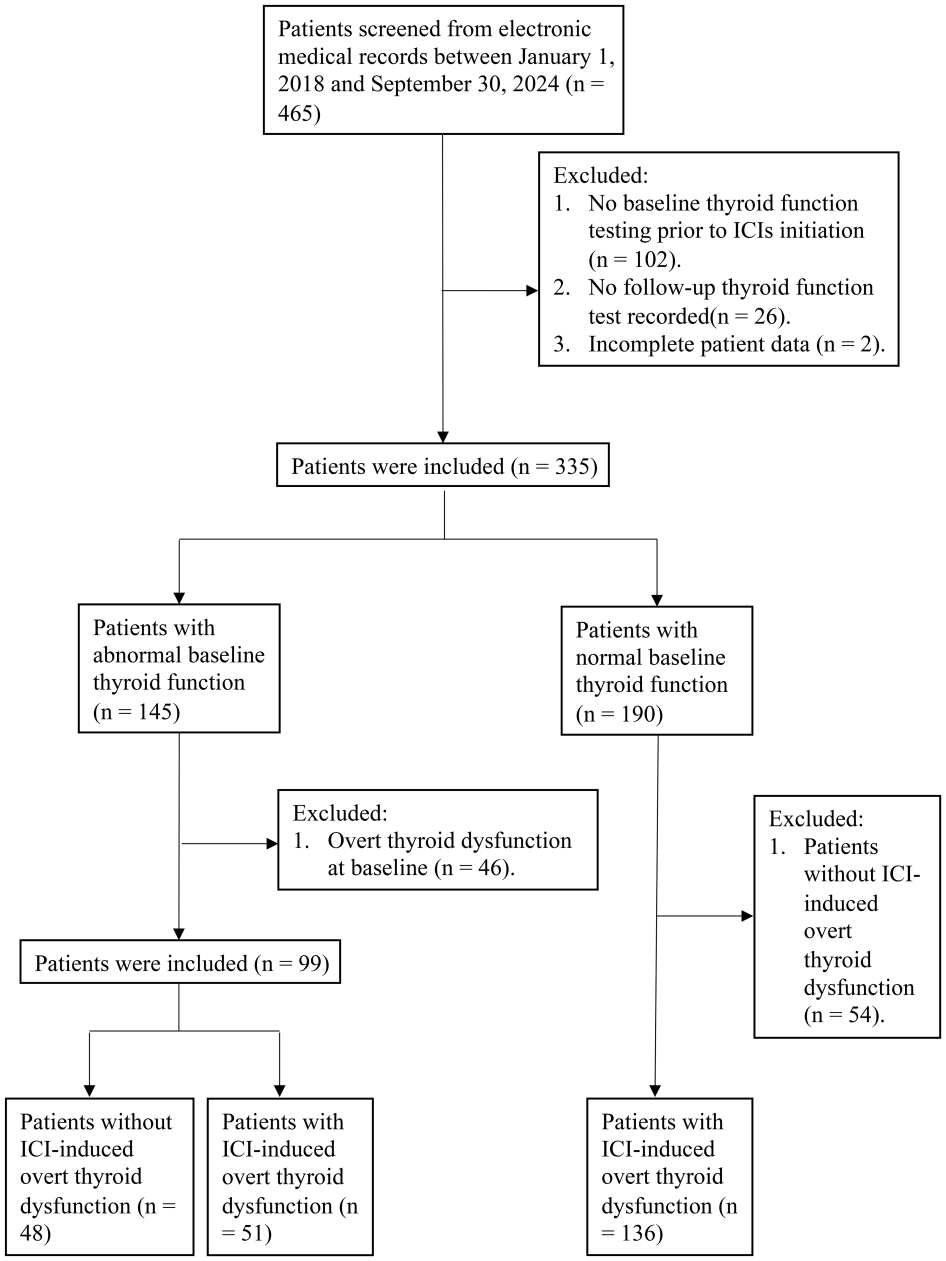
Flow diagram of patient selection.

After applying these criteria, 235 patients were selected for inclusion in the study. Among these, 99 had sABTF and 136 had NBTF with the subsequent development of ICI-induced overt thyroid dysfunction (**Figure 1**).

### 2.2 Definitions

ICI-induced overt thyroid dysfunction was defined as new-onset overt thyroid dysfunction that occurred after the initiation of ICI therapy as determined based on abnormal thyroid function test results. Overt thyrotoxicosis is defined as suppressed TSH levels accompanied by elevated FT4 and/or FT3 levels (9), while overt hypothyroidism is defined as elevated TSH levels with concurrently decreased FT4 levels (10). Subclinical thyrotoxicosis and hypothyroidism are characterized by abnormal TSH levels below or above the reference range, respectively—in the presence of normal FT4 concentrations (9, 11). Subclinical hypothyroidism was further stratified by severity: mild cases were defined as TSH levels above the reference range but below 10 mIU/L, whereas severe cases were defined as TSH levels of 10 mIU/L or higher (11). Isolated FT3/FT4 abnormality was defined as a baseline FT3 or FT4 concentration outside the laboratory reference interval with a concurrent TSH within the reference range.

The time of onset of overt thyroid dysfunction was defined as the interval between the initiation of ICI therapy and the date of the first thyroid function test showing overt dysfunction.

### 2.3 Laboratory measurements

Serum concentrations of FT3, FT4, and TSH were measured using an automated chemiluminescence immunoassay analyzer (Alinity; Abbott Laboratories; North Chicago, IL, USA) and Roche diagnostic kits (Roche Diagnostic; Roche Ltd.; Basel, Switzerland). Reference intervals established by the Peking University Cancer Hospital laboratory were as follows: FT3, 2.43–6.01 pmol/L; FT4, 9.01–19.05 pmol/L; and TSH, 0.35–4.94 mIU/L.

### 2.4 Covariates

The following covariates were extracted from the electronic medical records: demographic and lifestyle factors, including age, sex, height, weight, body mass index (BMI; calculated as weight in kilograms divided by height in meters squared), smoking history, and alcohol consumption history. The medical history variables included prior thyroid disease (hypothyroidism, thyrotoxicosis, hyperthyroidism, thyroiditis, or partial thyroidectomy), hypertension, diabetes mellitus, and previous exposure to ICIs. The clinical characteristics included the Eastern Cooperative Oncology Group performance status, tumor type and stage, line therapy, ICI treatment regimen (e.g., monotherapy or combination therapy), and whether radiotherapy was administered as part of the treatment course. The baseline laboratory parameters included albumin, serum creatinine, alanine aminotransferase, aspartate aminotransferase, absolute neutrophil, absolute lymphocyte, and absolute platelet counts. Neutrophil-to-lymphocyte ratio (NLR) and platelet-to-lymphocyte ratio were calculated.

### 2.5 Statistical analysis

Continuous variables were tested for normality using appropriate statistical tests. Variables with a normal distribution are reported as means ± standard deviations and were compared using independent-samples t-tests. Non-normally distributed variables are expressed as medians and interquartile ranges (IQRs), with between-group comparisons performed using the Mann-Whitney *U* test. Categorical variables are summarized as frequencies and percentages and were compared using Pearson’s chi-square test or Fisher’s exact test, as appropriate. Multivariate logistic regression analyses were conducted to identify the factors associated with outcomes, with covariates selected based on univariate *p*-values < 0.10 and clinical relevance. Odds ratios (ORs) and corresponding 95% confidence intervals (CIs) were reported.

To flexibly model the potential nonlinear association between the baseline FT4 levels and ICI-induced overt hypothyroidism, we employed restricted cubic spline (RCS) regression. FT4 was fitted with RCS functions using 4 knots located at equally spaced percentiles (5th, 35th, 65th, and 95th percentiles). Logistic regression models were constructed with RCS-transformed FT4 values as predictors, adjusting for potential confounders including sex, age, BMI, and NLR. The overall association and presence of nonlinearity were assessed using Wald chi-square tests, and *p*-values for both the overall and nonlinear effects were reported. Adjusted ORs and 95% CIs were plotted against the FT4 levels.

To determine the optimal cutoff value of FT4 for predicting the risk of ICI-induced overt hypothyroidism, receiver operating characteristic (ROC) curve analysis was performed. The Youden index (sensitivity + specificity − 1) was used to identify the optimal threshold that maximized the overall diagnostic accuracy. The area under the ROC curve was calculated to assess the discriminative ability of FT4.

Time-to-event outcomes were analyzed using Kaplan-Meier survival curves, and between-group comparisons were performed using the log-rank test. To identify the independent predictors of earlier onset, Cox proportional hazards models were constructed, adjusting for age and sex based on clinical relevance. Hazard ratios (HRs) and corresponding 95% CIs were reported.

All statistical tests were two-sided, and a *p*-value < 0.05 was considered statistically significant. Analyses were conducted using R version 4.2.2 (R Foundation for Statistical Computing, Vienna, Austria), and R packages “rms,” “pROC,” and “survival.” Figures were generated using GraphPad Prism, version 10 (GraphPad Software, San Diego, CA, USA).

## 3 Results

### 3.1 Risk of ICI-induced overt thyroid dysfunction in patients with pre-existing abnormal baseline thyroid function

Among the 99 patients with sABTF at baseline, 72 had subclinical hypothyroidism (46 mild, 26 severe), six had subclinical thyrotoxicosis, and 21 had isolated FT4/FT3 abnormalities. During ICI therapy, 51/99 (51.52%) developed ICI-induced overt thyroid dysfunction: 37 (37.37%) overt hypothyroidism and 14 (14.14%) overt thyrotoxicosis. By baseline subgroup, the incidence of overt thyroid dysfunction was 21/46 (45.65%) in mild subclinical hypothyroidism (17 overt hypothyroidism; four overt thyrotoxicosis), 17/26 (65.38%) in severe subclinical hypothyroidism (13 overt hypothyroidism; four overt thyrotoxicosis), 4/6 (66.67%) in subclinical thyrotoxicosis (two overt hypothyroidism; two overt thyrotoxicosis), and 9/21 (42.86%) in isolated FT4/FT3 abnormalities (five overt hypothyroidism; four overt thyrotoxicosis).

Compared with those who did not develop overt dysfunction, the affected patients had significantly lower baseline FT4 levels (median 12.20 [IQR, 10.25–15.71] vs. 14.14 [IQR, 11.75–17.25] pmol/L; *p* = 0.018) and NLR (2.60 [IQR, 1.85–3.52] vs. 3.31 [IQR, 2.37–4.33]; *p* = 0.049). BMI was also higher in the overt dysfunction group, although this difference did not reach statistical significance (25.22 [IQR, 21.33–26.94] vs. 22.41 [IQR, 21.19–24.69] kg/m²; *p* = 0.066). No other significant differences in the clinical or laboratory characteristics were observed between the two groups (**Table 1**).

**Table 1:** Baseline characteristics of patients with abnormal thyroid function at immune checkpoint inhibitor treatment initiation, stratified by the development of immune-related overt thyroid dysfunction.

Characteristic	Without overt thyroid dysfunction (n = 48)	With overt thyroid dysfunction (n = 51)	*P*-value
Sex, n (%)			0.633
Male	25 (52.08%)	29 (56.86%)	
Female	23 (47.92%)	22 (43.14%)	
Age, years	57.30 ± 14.12	57.69 ± 10.00	0.907
BMI, kg/m2	22.41 (21.19, 24.69)	25.22 (21.33, 26.94)	0.066
Medical history, n (%)			
Thyroid disease	18 (37.50%)	18 (35.29%)	0.820
Hypertension	19 (39.58%)	14 (27.45%)	0.201
Diabetes mellitus	11 (22.92%)	6 (11.76%)	0.141
Smoking history, n (%)	19 (39.58%)	19 (37.25%)	0.812
Alcohol consumption, n (%)	13 (27.08%)	16 (31.37%)	0.639
ECOG performance status, n (%)			0.670
0	31 (64.58%)	35 (68.63%)	
1–2	17 (35.42%)	16 (31.37%)	
Site of tumor, n (%)			0.129
Thoracic	16 (33.33%)	11 (21.57%)	
Digestive system	8 (16.67%)	17 (33.33%)	
Others	24 (50.00%)	23 (45.10%)	
Stage, n (%)			0.937
I–II	3 (6.25%)	3 (5.88%)	
III	9 (18.75%)	11 (21.57%)	
IV	36 (75.00%)	37 (72.55%)	
Line of therapy, n (%)			0.914
<3	39 (81.25%)	41 (80.39%)	
≥3	9 (18.75%)	10 (19.61%)	
Prior use of ICIs	8 (16.67%)	10 (19.61)	0.750
ICIs combination regimen, n (%)			0.375
Monotherapy	7 (14.58%)	4 (7.84%)	
CTLA-4	1 (2.08%)	1 (1.96%)	
Chemotherapy	24 (50.00%)	21 (41.18%)	
Targeted therapy	9 (18.75%)	18 (35.29%)	
Chemotherapy and targeted	7 (14.58%)	7 (13.73%)	
Radiotherapy, n (%)	4 (8.33%)	4 (7.84%)	>0.999
Baseline thyroid function abnormalities, n (%)			0.288
Mild subclinical hypothyroidism	25 (52.08%)	21 (41.18%)	
Severe subclinical hypothyroidism	9 (18.75%)	17 (33.33%)	
Subclinical thyrotoxicity	2 (4.17%)	4 (7.84%)	
FT4 or FT3 abnormality	12 (25.00%)	9 (17.65%)	
Baseline thyroid indicators			
TSH, mIU/L	5.79 (2.87, 7.93)	6.33 (4.51, 12.17)	0.220
FT3, pmol/L	4.02 (3.19, 4.49)	3.77 (3.29, 4.29)	0.355
FT4, pmol/L	14.14 (11.75, 17.25)	12.20 (10.25, 15.71)	**0.018**
Laboratory indicators			
Albumin, g/L	43.20 (39.95, 45.62)	44.10 (39.40, 46.00)	0.680
Serum creatinine, μmol/L	61.55 (54.00, 72.50)	66.00 (55.00, 79.50)	0.267
ALT, IU/L	14.55 (11.75, 20.00)	15.00 (11.00, 21.50)	0.850
AST, IU/L	19.00 (16.00, 24.50)	20.00 (15.50, 25.50)	0.744
NLR	3.31 (2.37, 4.33)	2.60 (1.85, 3.52)	**0.049**
PLR	156.76 (111.96, 218.22)	140.17 (108.16, 214.19)	0.619

Data are presented as mean ± standard deviation for continuous variables with normal distribution and as median (interquartile range) for non-normally distributed variables. Categorical variables are expressed as number (percentage). The normality of continuous variables was assessed using the Shapiro–Wilk test. Group comparisons were conducted using the independent-samples t-test for normally distributed variables and the Mann–Whitney U test for non-normally distributed variables. Categorical variables were compared using the Chi-square test or Fisher’s exact test, as appropriate. A two-sided p-value < 0.05 was considered statistically significant. Bold *p*-values indicate statistical differences.

BMI, body mass index; ECOG, Eastern Cooperative Oncology Group; ICI, immune checkpoint inhibitor; CTLA-4, cytotoxic T-lymphocyte-associated antigen 4; FT3, free triiodothyronine; FT4, free thyroxine; TSH, thyroid-stimulating hormone; ALT, alanine aminotransferase; AST, aspartate aminotransferase; NLR, neutrophil-to-lymphocyte ratio; PLR, platelet-to-lymphocyte ratio.

**Figure 2:**
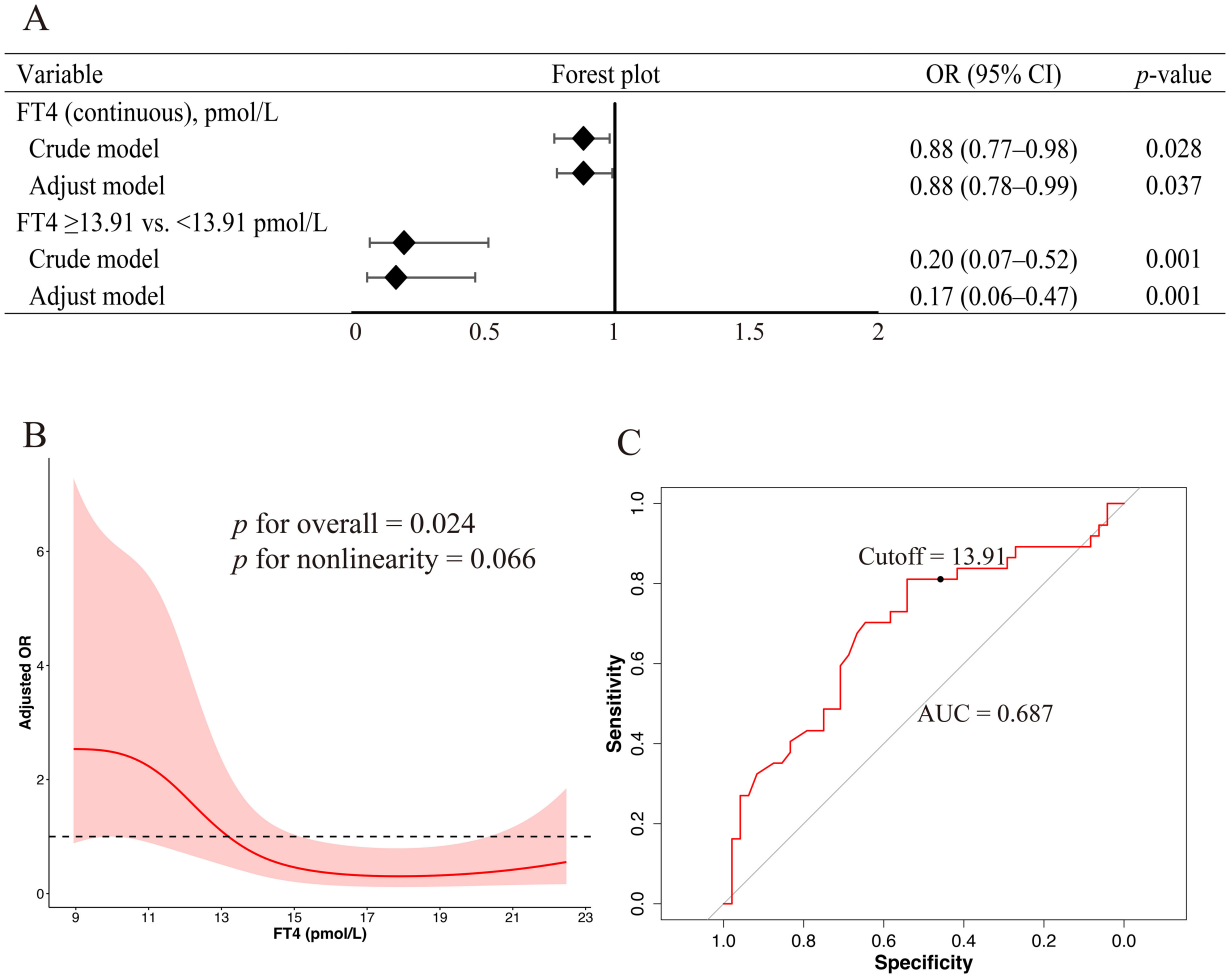
Association between baseline FT4 levels and the risk of ICI-induced overt hypothyroidism **(A)** Forest plots derived from logistic regression models evaluating FT4 as a continuous and categorical variable based on a 13.91 pmol/L cutoff. Variables included in the multivariable models were selected based on a univariate *p*-value < 0.10 and clinical relevance, including sex, age, BMI, and NLR. **(B)** RCS regression analysis revealed a potential nonlinear association between baseline FT4 levels and the risk of overt hypothyroidism. The solid red line indicates the adjusted OR and the shaded area represents the 95% CI. The model was adjusted for sex, age, BMI, and NLR. Four knots were placed at specified percentiles. **(C)** ROC curve of baseline FT4 levels for predicting overt hypothyroidism. FT4, free thyroxin; ICI, immune checkpoint inhibitor; BMI, body mass index; NLR, neutrophil-to-lymphocyte ratio; OR, odds ratio; RCS, restricted cubic spline; CI, confidence interval; ROC, receiver operating characteristic.

Multivariate logistic regression analysis was conducted to identify factors associated with the development of ICI-induced overt thyroid dysfunction. In the overall cohort, none of the examined variables, including age, sex, BMI, baseline FT4 level, and NLR, were significantly associated with the outcome (**Table 2**). However, the subgroup analysis revealed that lower baseline FT4 levels were associated with a higher risk of developing overt hypothyroidism (adjusted OR, 0.88; 95% CI, 0.78–0.99; *p* = 0.037; **Figure 2A**).

**Table 2:** Multivariate logistic regression analysis of risk factors for immune checkpoint inhibitor-induced thyroid dysfunction and its subtypes in patients with abnormal baseline thyroid function.

Characteristic	Thyroid dysfunction (n = 99)^1^		Hypothyroidism (n = 85)^1^		Thyrotoxicosis (n = 62)^1^	
	OR (95%CI)	*P*-value	OR (95%CI)	*P*-value	OR (95%CI)	*P*-value
Sex						
Male	1.00 (Reference)		1.00 (Reference)		1.00 (Reference)	
Female	0.82 (0.37–1.82)	0.633	0.67 (0.27–1.68)	0.396	1.11 (0.30–4.06)	0.873
Age, years	1.00 (0.97–1.04)	0.872	1.00 (0.97–1.04)	0.809	0.97 (0.92–1.03)	0.287
BMI, kg/m2	1.10 (0.98–1.23)	0.115	1.07 (0.94–1.22)	0.320	1.13 (0.92–1.39)	0.228
FT4, pmol/L	0.93 (0.85–1.02)	0.121	0.88 (0.78–0.99)	**0.037**	1.01 (0.88–1.16)	0.914
NLR	0.89 (0.75–1.07)	0.217	0.97 (0.81–1.16)	0.750	0.62 (0.36–1.07)	0.087

Multivariate logistic regression models were constructed to assess the association between selected variables and the development of ICI-induced overt thyroid dysfunction. Variables included in the models were those with p < 0.1 in univariate analyses and those deemed clinically relevant, including sex and age. The primary model compared patients who developed any form of ICI-induced overt thyroid dysfunction with those who did not. Subgroup analyses were additionally performed to separately evaluate risk factors for overt hypothyroidism and overt thyrotoxicosis.

^1^Models comprised the 48 patients with abnormal baseline thyroid function who did not develop overt thyroid dysfunction as the reference. Case counts were: 51 overt thyroid dysfunctions; 37 overt hypothyroidisms; 14 overt thyrotoxicosis. Bold *p*-values indicate statistical differences.

ICI, Immune checkpoint inhibitor; OR, odds ratio; CI, confidence interval; BMI, body mass index; FT4, free thyroxine; NLR, neutrophil-to-lymphocyte ratio.

### 3.2 Nonlinear association and predictive threshold of baseline FT4 in ICI-induced overt hypothyroidism

RCS regression was used to explore the potential nonlinear association between the baseline FT4 levels and risk of ICI-induced overt hypothyroidism. Baseline FT4 level was significantly associated with the outcome (*p* for overall association = 0.024); however, the test for nonlinearity did not reach statistical significance (*p* = 0.066), suggesting that the association may be approximately nonlinear (**Figure 2B**). ROC curve analysis demonstrated that baseline FT4 levels had a moderate discriminatory ability for predicting overt hypothyroidism, with an area under the ROC curve of 0.687. The optimal FT4 cutoff, determined using the maximum Youden index, was 13.91 pmol/L, corresponding to a sensitivity of 81.08% and specificity of 54.17% (**Figure 2C**). Subsequent logistic regression analysis using this threshold revealed that patients with baseline FT4 levels ≥13.91 pmol/L had a significantly lower risk of developing ICI-induced overt hypothyroidism than those below this threshold (adjusted OR, 0.17; 95% CI, 0.06–0.47; *p* = 0.001; **Figure 2A**).

### 3.3 Comparison of patients with abnormal and normal baseline thyroid function among those who developed ICI-induced overt thyroid dysfunction

Among the patients who developed ICI-induced overt thyroid dysfunction, those with sABTF (n = 51) were significantly more likely to have a history of thyroid disease than those with NBTF (35.29% vs. 11.76%; *p* < 0.001). Prior exposure to ICIs was also more common in the sABTF group (19.61% vs. 5.15%, *p* = 0.005). In addition, the incidence of overt hypothyroidism was significantly higher in the patients with sABTF than that in those with NBTF (72.55% vs. 53.68%, *p* = 0.020). Although the difference was not statistically significant, patients in the sABTF group showed a trend toward lower rates of smoking history (37.25% vs. 52.21%; *p* = 0.068) and were more likely to have received targeted therapy in combination with ICIs (35.29% vs. 19.12%; *p* = 0.059) (**Table 3**).

**Table 3:** Clinical characteristics of patients with immune checkpoint inhibitor-induced thyroid dysfunction, stratified by baseline thyroid function status.

Characteristic	Abnormal (n = 51)	Normal (n = 136)	*P*-value
Sex, n (%)			0.833
Male	29 (56.86%)	75 (55.15%)	
Female	22 (43.14%)	61 (44.85%)	
Age, years	57.69 ± 10.00	58.12 ± 10.59	0.802
BMI, kg/m2	25.22 (21.33, 26.94)	23.35 (21.20, 25.63)	0.179
Medical history, n (%)			
Thyroid disease	18 (35.29%)	16 (11.76%)	**<0.001**
Hypertension	14 (27.45%)	40 (29.41)	0.792
Diabetes mellitus	6 (11.76%)	19 (13.97%)	0.693
Smoking history, n (%)	19 (37.25%)	71 (52.21%)	0.068
Alcohol consumption, n (%)	16 (31.37%)	41 (30.15%)	0.871
ECOG performance status, n (%)			0.441
0	35 (68.63%)	101 (74.26%)	
1–2	16 (31.37%)	35 (25.74%)	
Site of tumor, n (%)			0.957
Thoracic	11 (21.57%)	32 (23.53%)	
Digestive system	17 (33.33%)	45 (33.09%)	
Others	23 (45.10%)	59 (43.38%)	
Stage, n (%)			0.588
I–II	3 (5.88%)	5 (5.68%)	
III	11 (21.57%)	26 (29.55%)	
IV	37 (72.55%)	57 (64.77%)	
Line of therapy, n (%)			0.460
<3	41 (80.39%)	75 (85.23%)	
≥3	10 (19.61%)	13 (14.77%)	
Prior use of ICIs	10 (19.61%)	5 (5.15%)	**0.005**
ICIs combination regimen, n (%)			0.059
Monotherapy	4 (7.84%)	21 (15.44%)	
CTLA-4	1 (1.96%)	2 (1.47%)	
Chemotherapy	21 (41.18%)	76 (55.88%)	
Targeted therapy	18 (35.29%)	26 (19.12%)	
Chemotherapy and targeted	7 (13.73%)	11 (8.09%)	
Radiotherapy, n (%)	4 (7.84%)	7 (5.15%)	0.727
Laboratory indicators			
Albumin, g/L	44.10 (39.40, 46.00)	44.20 (42.00, 46.50)	0.228
Serum creatinine, μmol/L	66.00 (55.00, 79.50)	62.00 (53.00, 72.00)	0.129
ALT, IU/L	15.00 (11.00, 21.50)	16.00 (11.00, 22.00)	0.687
AST, IU/L	20.00 (15.50, 25.50)	18.00 (15.00, 23.00)	0.196
NLR	2.60 (1.85, 3.52)	2.72 (1.84, 4.22)	0.374
PLR	140.17 (108.16, 214.19)	165.59 (120.84, 236.65)	0.218
Outcome			**0.020**
Overt thyrotoxicosis	14 (27.45%)	73 (53.68%)	
Overt hypothyroidism	37 (72.55%)	63 (46.32%)	

Data are presented as mean ± standard deviation for continuous variables with normal distribution and as median (interquartile range) for non-normally distributed variables. Categorical variables are expressed as number (percentage). The normality of continuous variables was assessed using the Shapiro–Wilk test. Group comparisons were conducted using the independent-samples t-test for normally distributed variables and the Mann–Whitney U test for non-normally distributed variables. Categorical variables were compared using the Chi-square test or Fisher’s exact test, as appropriate. A two-sided p-value < 0.05 was considered statistically significant. Bold *p*-values indicate statistical differences.

ICI, immune checkpoint inhibitor; BMI, body mass index; ECOG, Eastern Cooperative Oncology Group; CTLA-4, cytotoxic T-lymphocyte-associated antigen 4; ALT, alanine aminotransferase; AST, aspartate aminotransferase; NLR, neutrophil-to-lymphocyte ratio; PLR, platelet-to-lymphocyte ratio.

Logistic regression analysis was performed to identify the factors associated with sABTF- and ICI-induced overt thyroid dysfunction among the patients. A history of thyroid disease was significantly associated with increased risk of sABTF (OR, 4.09; 95% CI, 1.88–8.89; *p* < 0.001). Similarly, previous exposure to ICIs was associated with sABTF (OR, 4.49; 95% CI, 1.61–12.56; *p* = 0.004). Among the patients with sABTF, those who received ICIs in combination with targeted therapy had a significantly higher risk of developing overt thyroid dysfunction than those treated with ICIs alone (OR, 3.63; 95% CI, 1.07–12.39; *p* = 0.039). Additionally, among the patients who developed overt thyroid dysfunction, those who progressed to hypothyroidism were more likely to have had abnormal thyroid function at baseline than those who developed thyrotoxicosis (OR, 2.28; 95% CI, 1.13–4.60; *p* = 0.021) (**Table 4**).

**Table 4:** Multivariate logistic regression analysis of factors associated with abnormal baseline thyroid function in patients who developed immune checkpoint inhibitor-induced thyroid dysfunction.

Characteristic	OR (95%CI)	*P*-value
Sex		
Male	1.00 (Reference)	
Female	0.93 (0.49–1.78)	0.833
Age, years	1.00 (0.97–1.03)	0.800
BMI, kg/m2	1.03 (0.94–1.13)	0.495
History of thyroid disease		
No	1.00 (Reference)	
Yes	4.09 (1.88–8.89)	**<0.001**
Smoking history		
No	1.00 (Reference)	
Yes	0.54 (0.28–1.05)	0.070
Prior use of ICIs		
No	1.00 (Reference)	
Yes	4.49 (1.61–12.56)	**0.004**
ICIs combination regimen		
Monotherapy	1.00 (Reference)	
CTLA-4	2.63 (0.19–36.34)	0.472
Chemotherapy	1.45 (0.45–4.69)	0.534
Targeted therapy	3.63 (1.07–12.39)	**0.039**
Chemotherapy and targeted	3.34 (0.80–13.94)	0.098
Outcome		
Thyrotoxicosis	1.00 (Reference)	
Hypothyroidism	2.28 (1.13–4.60)	**0.021**

ORs and 95% CIs were calculated using multivariate logistic regression. Variables included in the models were those with p < 0.1 in univariate analyses and those deemed clinically relevant, including sex age, and BMI. A two-sided p-value < 0.05 was considered statistically significant. Bold *p*-values indicate statistical differences.

Abbreviations: ICI, immune checkpoint inhibitor; OR, odds ratio; CI, confidence interval; BMI, body mass index; CTLA-4, cytotoxic T-lymphocyte-associated antigen 4.

### 3.4 Time-to-event analysis of ICI-induced overt thyroid dysfunction

**Figure 3:**
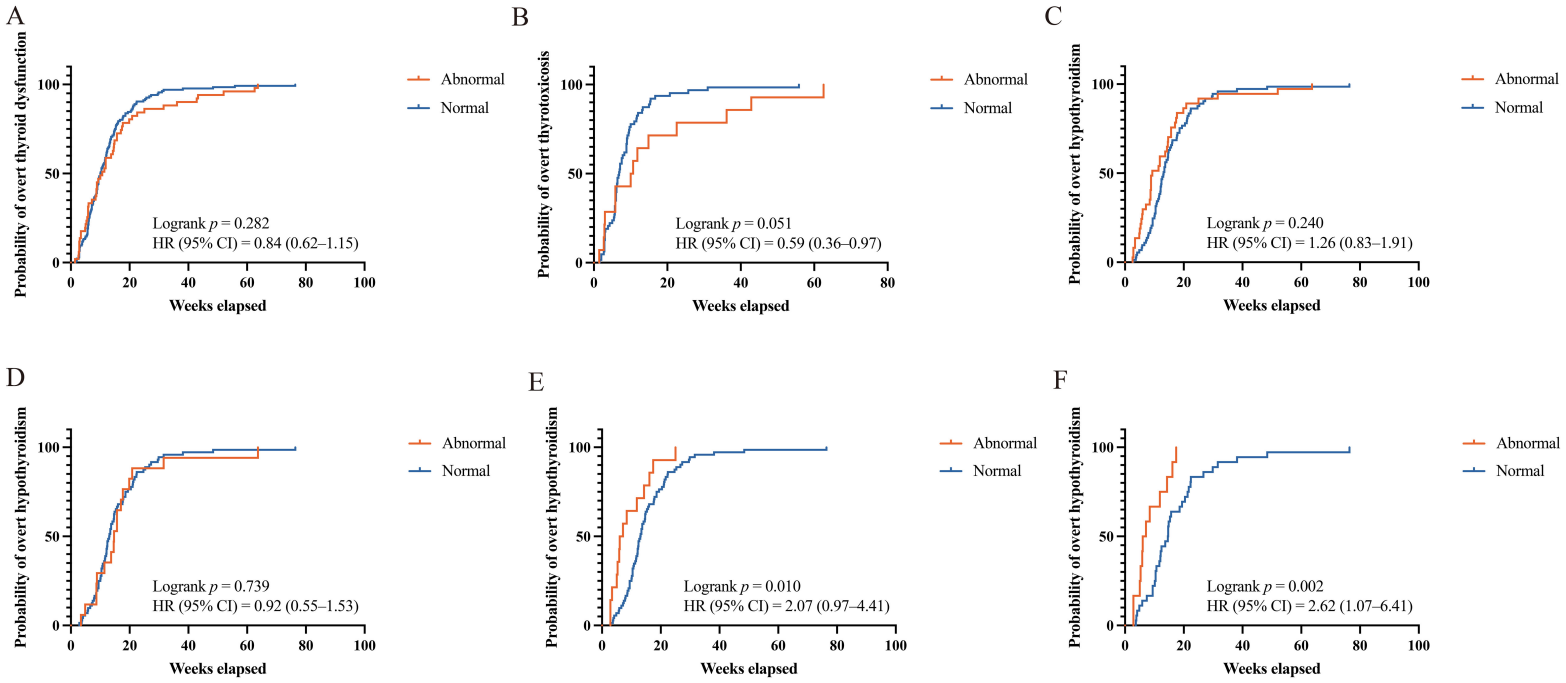
Kaplan-Meier curves of time to ICI-induced overt thyroid dysfunction. **(A)** Any overt thyroid dysfunction, abnormal versus normal baseline thyroid function. **(B)** Overt thyrotoxicosis, abnormal versus normal baseline thyroid function. **(C)** Overt hypothyroidism, abnormal versus normal baseline thyroid function. **(D)** Overt hypothyroidism, mild subclinical hypothyroidism (baseline TSH level above the reference range but <10 mIU/L) versus those with normal baseline thyroid function. **(E)** Overt hypothyroidism, severe subclinical hypothyroidism (baseline TSH ≥10 mIU/L) versus those with normal baseline thyroid function. **(F)** Overt hypothyroidism, severe subclinical hypothyroidism (baseline TSH ≥10 mIU/L) and baseline FT4 <13.91 pmol/L versus those with normal baseline thyroid function. HR, hazard ratios; CI, confidence interval; ICI, immune checkpoint inhibitor; TSH, thyroid-stimulating hormone; FT4, free thyroxine.

We compared the time to onset of ICI-induced overt thyroid dysfunction between the patients with sABTF and those with NBTF. The median time to onset was comparable between the groups (10.00 weeks [IQR, 5.71–16.36], sABTF group; 10.07 weeks [IQR, 6.25–15.07], NBTF group). In the subgroup analyses, the median time to overt thyrotoxicosis was shorter in the patients with NBTF (6.86 weeks [IQR, 5.50–9.64]) than that in those with sABTF (10.36 weeks [IQR, 3.71–20.64]). In contrast, the median time to overt hypothyroidism was longer in the NBTF group (13.14 weeks [IQR, 10.00–18.57]) than that in the sABTF group (9.29 weeks [IQR, 5.86–15.71]). However, these differences were not statistically significant (**Figure 3**).

Among the patients with baseline subclinical hypothyroidism, 17 with mild TSH elevation (TSH above the upper limit of normal but <10 mIU/L) and 13 with severe TSH elevation (TSH ≥10 mIU/L) progressed to overt hypothyroidism during ICI therapy. Compared with patients with NBTF, those with severe subclinical hypothyroidism experienced a significantly shorter median time to overt hypothyroidism (7.78 weeks [IQR, 4.61–16.46; HR, 2.07; 95% CI, 0.97–4.41; *p* = 0.010]). Notably, when severe subclinical hypothyroidism was accompanied by a baseline FT4 level <13.91 pmol/L, the median time to overt hypothyroidism was even shorter with a more pronounced and statistically significant risk elevation. The median time to overt hypothyroidism was 6.57 weeks (IQR, 5.07–13.68; HR, 2.62; 95% CI, 1.07–6.41; *p* = 0.002). In contrast, no statistically significant difference in the time to overt hypothyroidism was observed between the patients with mild subclinical hypothyroidism and those with NBTF (**Figure 3**).

To further assess the impact of baseline thyroid status, a Cox proportional hazards model was used to compare the patients with severe subclinical hypothyroidism to those with NBTF. In the fully adjusted model, severe subclinical hypothyroidism remained associated with a significantly increased risk of developing overt hypothyroidism (adjusted HR, 2.84; 95% CI, 1.42–5.67; *p* = 0.003). The association was even more pronounced in the patients with severe subclinical hypothyroidism and concurrent FT4 <13.91 pmol/L (adjusted HR, 10.05; 95% CI, 3.13–32.23; *p* < 0.001) (**Table 5**).

**Table 5:** Multivariate Cox proportional hazards models evaluating time to overt hypothyroidism in patients with severe subclinical hypothyroidism versus those with normal baseline thyroid function.

Model	TSH > 10 mIU/L		TSH > 10 mIU/L and FT4 <13.91 pmol/L	
	HR (95%CI)	*P*-value	HR (95%CI)	*P*-value
Model 1	2.36 (1.30–4.31)	**0.005**	2.95 (1.45–6.01)	**0.003**
Model 2	2.32 (1.27–4.24)	**0.006**	2.98 (1.41–6.30)	**0.004**
Model 3	2.84 (1.42–5.67)	**0.003**	10.05 (3.13–32.23)	**<0.001**

HRs and 95% CIs were estimated using Cox proportional hazards regression. Model 1: unadjusted model. Model 2: adjusted for age (continuous), sex (male or female), and BMI (continuous). Model 3: further adjusted for history of thyroid disease (yes or no), smoking history (yes or no), prior use of ICIs (yes or no), and ICI combination regimen (monotherapy, CTLA-4 inhibitor, chemotherapy, targeted therapy, or chemotherapy plus targeted therapy). A two-sided p-value < 0.05 was considered statistically significant. Bold *p*-values indicate statistical differences.

HR, hazard ratio; CI, confidence interval; BMI: body mass index; ICI, immune checkpoint inhibitor; CTLA-4, cytotoxic T-lymphocyte-associated antigen 4.

## 4 Discussion

We conducted this retrospective cohort study to characterize the clinical course of ICI-induced overt thyroid dysfunction in a population of patients with cancer and sABTF. We found that preexisting sABTF was associated with both the risk and timing of overt thyroid dysfunction during immunotherapy. Patients with NBTF who did not develop overt dysfunction were excluded by design; therefore, this study could not estimate the incidence in the overall NBTF population. Previous studies have estimated the overall incidence of ICI-induced overt thyroid dysfunction as approximately 20% in both Western and Chinese populations (3, 4, 12). Importantly, the present study revealed that nearly half of the patients with sABTF progressed to overt thyroid dysfunction during ICI therapy, which is substantially higher than that reported in previous studies. These findings support the need for enhanced surveillance and proactive management of this patient population.

A key finding of our study is that baseline FT4 levels may serve as predictive markers for ICI-induced overt hypothyroidism, particularly in patients with pre-existing sABTF. The RCS analysis demonstrated a significant overall association between baseline FT4 levels and the risk of subsequent overt hypothyroidism, with a non-significant trend toward nonlinearity, suggesting a potential threshold effect. Consistent with this analysis, the ROC curve analysis identified an optimal FT4 cutoff of 13.91 pmol/L for risk discrimination. Supporting this observation, logistic regression analysis showed that the patients with baseline FT4 ≥13.91 pmol/L had an 83% reduction in the odds of developing overt hypothyroidism compared with that in those below the cutoff (adjusted OR, 0.17; 95% CI, 0.06–0.47; *p* = 0.001).

Collectively, these findings address a notable gap in the risk stratification of ICI-induced overt hypothyroidism. Previous studies have identified elevated baseline TSH as a key predictor of subsequent thyroid dysfunction in patients who are euthyroid at the initiation of ICI therapy (13–15). However, in individuals with preexisting sABTF, such as those with isolated TSH elevation or suppression, baseline TSH is already outside the reference range; therefore, this risk factor loses its predictive utility. Our results indicate that baseline FT4 serves as a more reliable biomarker for predicting the risk of overt hypothyroidism in this subset of patients. These findings have important clinical implications. Risk stratification based on baseline FT4 levels can facilitate the identification of high-risk individuals, enabling more intensive monitoring and timely intervention. Such an approach may support personalized management of thyroid function during ICI therapy.

We found that a history of thyroid disease was strongly associated with sABTF in patients who developed ICI-induced overt thyroid dysfunction. This finding is consistent with our expectations, as preexisting thyroid disorders, particularly autoimmune thyroiditis, are often characterized by chronic hormonal dysregulation or require ongoing thyroid hormone replacement therapy, both of which can result in sABTF. Mechanistically, patients with a history of thyroid disease likely enter immunotherapy with a pre-existing disruption of the hypothalamic–pituitary–thyroid axis, rendering them more susceptible to additional immune-mediated thyroid damage upon ICI exposure. This aligns with the existing evidence showing that underlying thyroid autoimmunity increases vulnerability to ICI-induced thyroid dysfunction (16, 17). In addition, prior exposure to ICIs was associated with sABTF, likely reflecting residual or irreversible thyroid injury caused by previous irAEs. Given that thyroid irAEs frequently result in permanent hypothyroidism, patients who restart ICI therapy may present with persistently abnormal thyroid profiles. Patients undergoing multiple courses of or sequential immunotherapy regimens may be particularly prone to cumulative endocrine toxicity. Collectively, these findings highlight the importance of thoroughly assessing both thyroid disease history and immunotherapy exposure before initiating a new course of ICIs. This information can inform individualized monitoring strategies, particularly for patients with the potential of preexisting thyroid injuries.

Moreover, of the patients with sABTF, those receiving ICIs in combination with targeted therapies exhibited a significantly higher risk of developing ICI-induced overt thyroid dysfunction than that in those receiving ICIs alone. These findings suggest a potential additive or synergistic effect on thyroid toxicity. Notably, many targeted agents—particularly tyrosine kinase inhibitors—are known to disrupt thyroid function (18, 19). Furthermore, previous studies have reported that combining targeted therapies with ICIs may further increase the incidence of thyroid irAEs (20). Therefore, clinicians should be vigilant of the patients with sABTF who are receiving concomitant targeted drug therapy and alert these individuals to the potential high-risk of thyroid dysfunction. Baseline screening and periodic thyroid function tests should be conducted before patients initiate ICI treatment.

From a clinical management perspective, our findings highlight the importance of comprehensive baseline thyroid function assessment, including both TSH and FT4 levels, prior to initiating ICI therapy in patients. In particular, patients with severe subclinical hypothyroidism at baseline (TSH ≥10 mIU/L) were at significantly increased risk for rapid progression to overt hypothyroidism following ICI initiation, with a median onset time of 7.78 weeks (IQR, 4.61–16.46), notably shorter than that observed in euthyroid individuals. Even after adjustment, a baseline TSH ≥10 mIU/L was associated with a greater than two-fold increase in the hazard of developing overt hypothyroidism. Furthermore, in patients with severe subclinical hypothyroidism accompanied by FT4 <13.91 pmol/L, the progression occurred even earlier, with the risk increasing up to tenfold. These findings emphasize the need for increased clinical vigilance in this subgroup, warranting frequent thyroid function monitoring to enable early detection and prompt initiation of hormone replacement therapy. Multidisciplinary management involving endocrinology specialists should be considered to optimize patient outcomes.

This study had several limitations that warrant consideration when interpreting the results. First, unmeasured confounding factors were present owing to thyroid autoimmunity. We did not systematically assess thyroid autoantibodies (e.g., anti-thyroid peroxidase and anti-thyroglobulin), which are plausibly associated with both exposure and outcome. Although major guidelines recommend pre-ICI thyroid function testing, routine autoantibody screening is not mandatory. Moreover, antibody testing is not routinely performed in our oncology practice. Consequently, we could not evaluate or adjust for this potential confounder. Second, the retrospective design led to non-standardized testing intervals, which may have introduced discrepancies between the actual onset and documented diagnosis of thyroid dysfunction. Third, the sample sizes of certain subgroups, particularly those including patients with severe subclinical hypothyroidism, were small. Finally, some patients may have initiated thyroid hormone replacement therapy before ICI therapy. Furthermore, our institution does not have an in-house endocrinology service; therefore, many patients receive thyroid care at outside facilities. As a result, treatment initiation and dose adjustments were not systematically captured in our analysis. Prospective studies with external validation cohorts are needed to confirm our findings.

In summary, this study demonstrated that preexisting sABTF significantly influences the risk and timing of ICI-induced overt hypothyroidism. Baseline FT4 emerged as a valuable predictive biomarker, with a clinically relevant threshold of 13.91 pmol/L identified for risk stratification. Severe subclinical hypothyroidism notably accelerated the onset of overt hypothyroidism. When accompanied by FT4 <13.91 pmol/L, patients not only faced a markedly higher risk but also experienced earlier onset, representing a high-priority subgroup for clinical attention. Furthermore, a history of thyroid disease and prior ICI exposure were associated with a greater likelihood of sABTF. These findings highlight the importance of obtaining a comprehensive medical history and conducting a thorough baseline thyroid function assessment, including both TSH and FT4 levels, before the initiation of ICI therapy. Future studies are warranted to validate these observations and refine monitoring protocols for early detection and timely intervention.

## Data Availability

The original contributions presented in the study are included in the article/supplementary material. Further inquiries can be directed to the corresponding author.
